# Lipid peroxidation causes endosomal antigen release for cross-presentation

**DOI:** 10.1038/srep22064

**Published:** 2016-02-24

**Authors:** Ilse Dingjan, Daniëlle RJ Verboogen, Laurent M Paardekooper, Natalia H Revelo, Simone P Sittig, Linda J Visser, Gabriele Fischer von Mollard, Stefanie SV Henriet, Carl G Figdor, Martin ter Beest, Geert van den Bogaart

**Affiliations:** 1 Department of Tumor Immunology, Radboud Institute for Molecular Life Sciences, Radboud University Medical Center, Nijmegen, 6525 GA, the Netherlands; 2 Department of Chemistry, Bielefeld University, Bielefeld, 33501, Germany; 3 Department of Pediatrics, Radboud University Medical Center, Nijmegen, 6525 GA, the Netherlands

## Abstract

Dendritic cells (DCs) present foreign antigen in major histocompatibility complex (MHC) class I molecules to cytotoxic T cells in a process called cross-presentation. An important step in this process is the release of antigen from the lumen of endosomes into the cytosol, but the mechanism of this step is still unclear. In this study, we show that reactive oxygen species (ROS) produced by the NADPH-oxidase complex NOX2 cause lipid peroxidation, a membrane disrupting chain-reaction, which in turn results in antigen leakage from endosomes. Antigen leakage and cross-presentation were inhibited by blocking ROS production or scavenging radicals and induced when using a ROS-generating photosensitizer. Endosomal antigen release was impaired in DCs from chronic granulomatous disease (CGD) patients with dysfunctional NOX2. Thus, NOX2 induces antigen release from endosomes for cross-presentation by direct oxidation of endosomal lipids. This constitutes a new cellular function for ROS in regulating immune responses against pathogens and cancer.

Dendritic cells (DCs) are antigen presenting cells of the immune system capable to activate naive T cells with exogenous antigen^1^,^2^,^3^. DCs constantly sample peripheral tissues for microbial pathogens and tumor cells that can be taken up by phago- or endocytosis. For activation of CD4 + T helper cells, these antigens are subsequently degraded in lysosomal compartments and presented in MHC class II. For activation of CD8 + cytotoxic T cells, exogenous antigens need to be presented in MHC class I in a process called cross-presentation. Cross-presentation can occur via a lysosomal and a cytosolic pathway. In the lysosomal pathway, antigen is degraded by lysosomal proteases and loaded in MHC class I in the lysosomal lumen. In the cytosolic pathway, antigen escapes from the endosomal lumen into the cytosol where it is processed by the proteasome for presentation in MHC class I^1^,^2^,^3^,^4^. Despite intensive research efforts, it is still unclear how antigen crosses the membrane of endosomes. The translocon Sec61^5^,^6^,^7^ from the endoplasmic reticulum associated degradation (ERAD) pathway has been shown to transport proteins from endosomes into the cytosol, but the interpretations as well as functional consequences of these studies were controversial^1^,^2^,^3^,^8^. This discussion was recently settled in an elegant study where they showed that the trapping of Sec61 in the endoplasmic reticulum with a single chain antibody blocked the release of antigen from endosomes and impaired cross-presentation^8^. However, this may not suffice to explain how large, folded and/or posttranslationally modified proteins as well as nucleic acids and dextrans are released from endosomes^3^,^9^ and, as also acknowledged in the study by Zehner *et al.*^8^, likely other pathways exist by which antigen is released from endosomes.

Antigen cross-presentation is promoted by the activity of the NADPH oxidase NOX2^10^,^11^,^12^,^13^,^14^. Already within 30 minutes after antigen uptake, NOX2 generates superoxide anions in the endosomal lumen in DCs and this production is sustained over the course of hours^10^,^11^,^12^,^13^,^14^,^15^,^16^. Superoxide anions react with protons resulting in the formation of H_2_O_2_ and alkalinization of the endosomal lumen. Thereby, NOX2 counteracts the activity of the V-ATPase and this inhibits the activation of lysosomal proteases with low pH optima, hence preserving the antigen for cross-presentation^10^,^11^,^12^. H_2_O_2_ and other reactive oxygen species (ROS) produced by NOX2 activity can also oxidatively inactivate cystein cathepsins, which further inhibits degradation of antigen in endosomes of DCs^14^,^16^,^17^. ROS also react with membranes and can oxidize membrane proteins, cholesterol and particularly (poly)unsaturated lipids in a process called lipid peroxidation^18^. Lipid peroxidation is a self-propagating chain reaction that proceeds very effectively, especially since oxygen is an apolar molecule which accumulates ~5-fold in lipid membranes compared to the aqueous environment^19^. Oxidized lipids have peroxide or aldehyde groups in their lipid tails, are more polar and can be shorter in length. As a consequence, lipid peroxidation is a destructive process, which is well-understood for over four decades to cause leakage of artificial and cellular membranes (see^20^,^21^,^22^ and references therein). Whereas leakage of endo/lysosomal contents to the cytosol may trigger cell death, this does not necessarily has to be the case^3^ and endosomal disruption is actually a widespread approach in the gene therapy research field for cellular delivery of nucleic acids^23^,^24^,^25^,^26^. In fact, endosomal disruption can be an efficient way to introduce antigen into the MHC class I pathway^9^,^27^,^28^. Therefore, we hypothesized that the sustained ROS produced in endosomes by NOX2 would directly cause release of antigens into the cytosol via lipid peroxidation-induced membrane destabilization.

## Results

### NOX2-produced ROS cause endosomal lipid peroxidation

**Figure 1:**
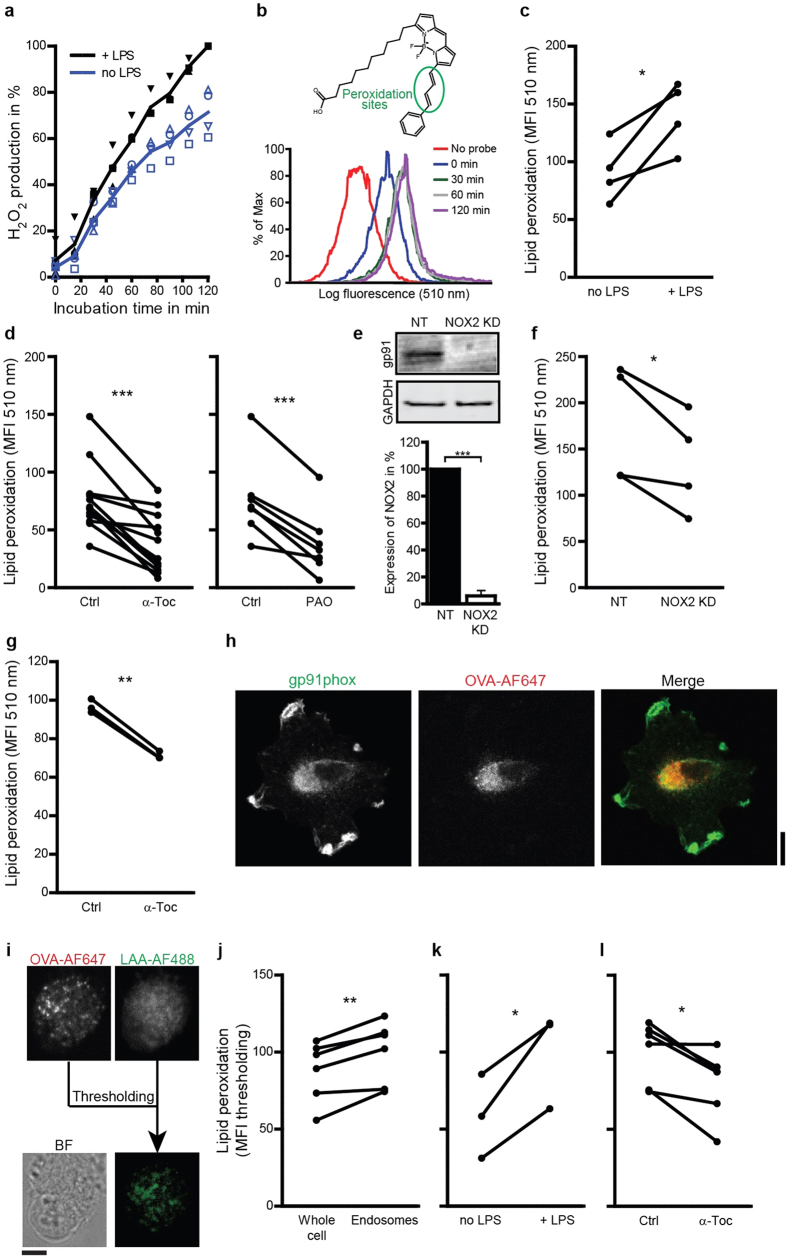
NOX2-produced ROS cause endosomal lipid peroxidation. (**a**) H_2_O_2_ production by DCs cultured in absence (no LPS; blue curve) or presence of LPS (+LPS; black) measured with Amplex Red. (**b**) Sensor Bodipy581/591-C11 (structure) increases fluorescence at 510 nm upon peroxidation. Histograms show fluorescence intensity distributions from a typical experiment of DCs labeled with Bodipy581/591-C11 and incubation with LPS for the times indicated. (**c–d**) The mean fluorescence intensities (MFI) measured by flow cytometry of DCs cultured for 60 min with Bodipy581/591-C11 in presence or absence of LPS (**c**), or with LPS in absence (Ctrl) or presence of α-tocopherol (α-Toc) or phenylarsine oxide (PAO) (**d**). (**e**) Quantification of knock-down efficiency for gp91phox (NOX2^KD^) by Western blot (NT: non-targeting siRNA; GAPDH: loading control). (**f**) Similar to c, but now for NOX2^KD^ DCs. (**g**) Similar to d, but now for mouse BMDCs. (**h**) Confocal microscope images of DCs stained for gp91phox (green) after treatment with OVA conjugated with Alexa fluor 647 (OVA-AF647; red). (**i**) Detection of peroxidation-modified proteins on endosomes. DCs were incubated with OVA-AF647 and linoleamide alkyne (LAA). After incubation, cells were fixed and stained with Alexa fluor 488 azide (LAA-AF488). The fluorescence threshold was set on the OVA-AF647 channel and the green fluorescence was quantified. BF: bright field. (**j**) Difference between green fluorescence of the whole cell and the fluorescence thresholded on OVA-AF647. (**k**) LAA assay for DCs cultured in the absence or presence of LPS. LAA-AF488 signal was quantified for at least 10 cells. (**l**) Same as panel (**k**), but now for LPS-treated DCs in the absence or presence of α-Toc. Results show individual results for at least 3 donors/mice. Results from the same donor/mouse are connected by a black line. Scale bars, 10 μm.

We first verified that ROS produced by NOX2 could trigger lipid peroxidation. We activated DCs with the TLR4-ligand LPS, which is well known to induce NOX2 activity^29^,^30^, resulting in an increase in ROS production (Fig. 1a). ROS can cause lipid peroxidation, which can be measured with the Bodipy581/591-C11 probe. Bodipy581/591-C11 contains alkene groups mimicking unsaturated fatty acids and making it susceptible to peroxidation^31^. This probe incorporates into cellular membranes and peroxidation causes a blue-shift of its fluorescence over time, which can be measured by flow cytometry (Fig. 1b). Directly oxidizing lipids with cumene hydroperoxide as a positive control resulted in a >3-fold increase of the blue fluorescence. Culturing the cells in the presence of LPS resulted in a ~50% increase in Bodipy581/591-C11 fluorescence (Fig. 1c) supporting a role for NOX2 in lipid peroxidation. Indeed, inhibition of NOX2 with the inhibitor phenylarsine oxide (PAO) decreased lipid peroxidation (Fig. 1d), although this compound is not specific for NOX2 as it oxidizes all vicinal thiols. We could not use the (more specific) NOX2-inhibitor diphenyleneiodonium (DPI), as this compound was too toxic for our DCs. To confirm the role of NOX2-produced ROS in lipid peroxidation, we measured peroxidation in human DCs which were siRNA silenced (RNAi) for gp91phox, one of the membrane components of NOX2 (NOX2^KD^; 94% knock-down efficiency; Fig. 1e)^32^. In NOX2^KD^ DCs, we found a ~25% reduction of Bodipy581/591-C11 signal (Fig. 1f), which was significant but relatively small, likely due to residual NOX activity^15^. These data indicate that NOX2 can induce lipid peroxidation in DCs.

To further confirm the role of ROS in lipid peroxidation, we used vitamin E. Vitamin E is a group of lipid soluble antioxidants that selectively block lipid peroxidation by scavenging free electrons^18^. In agreement, the most active form of vitamin E, α-tocopherol, decreased peroxidation of Bodipy581/591-C11 by ~50% (Fig. 1d). We did not expect that α-Tocopherol affects endosomal alkanization by sequestering ROS in the endosomal lumen, because it predominantly incorporates in membranes. This was confirmed by experiments with the pH-sensitive fluorophores SNARF-1 conjugated to dextran and pHrodo-Green conjugated to OVA (Suppl. Fig. 1; compare to ref. 13). We also performed the Bodipy581/591-C11 assay with mouse bone marrow-derived DCs (BMDCs) in combination with α-tocopherol treatment. Similar to human DCs, lipid peroxidation in BMDCs was decreased after α-tocopherol treatment (Fig. 1g). Immunostaining human DCs for gp91phox showed that NOX2 localized to the peripheral cell membrane and to endosomal compartments (Fig. 1h), suggesting that substantial peroxidation occurred in endosomal membranes. To address this, we employed the linoleamide alkyne (LAA) assay, where LPS-activated DCs were cultured in the presence of LAA which incorporates in cellular membranes. Peroxidation of LAA produces reactive aldehydes that indiscriminately bind to proteins and these modified proteins can be detected with Alexa fluor 448 azide (click chemistry)^33^. By pre-exposing the cells with OVA conjugated to Alexa fluor 647, we could distinguish peroxidation of endosomal membranes from membranes of other organelles (Fig. 1i). Indeed, endosomes (containing Alexa fluor 647-labeled OVA) were ~20% more peroxidated than other cellular membranes (Fig. 1j). Moreover, activating the cells with LPS increased lipid peroxidation in endosomal membranes ~1.7-fold (Fig. 1k) and peroxidation could again be inhibited with α-tocopherol (Fig. 1l), in accordance with our results with Bodipy581/591-C11 and further supporting a role for NOX2 in lipid peroxidation in endosomes of DCs. We then determined whether NOX2-induced lipid peroxidation could cause the release of antigen from endosomes.

### Lipid peroxidation causes antigen release from endosomes

**Figure 2:**
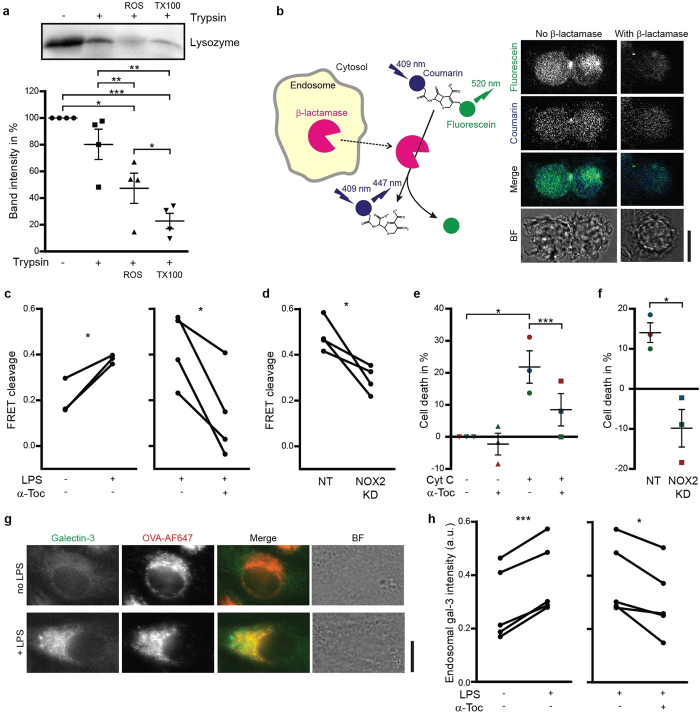
Lipid peroxidation causes antigen release from endosomes. (**a**) Liposomes with lysozyme encapsulated were treated with a combination of H_2_O_2_ and Fe(II)SO_4_ (ROS) or Triton X-100 (TX100; positive control) followed by incubation with trypsin. Samples were analyzed by SDS-PAGE. Graph shows semi-quantifications of band intensities from 4 independent experiments (mean ± S.E.M). (**b**) Scheme and confocal microscope images of a CCF4 assay. The cytosolic FRET probe CCF4 was cleaved by exogenous β-lactamase (β-lac) resulting in a decreased ratio of fluorescein (acceptor fluorophore; green) over coumarin (donor; blue) fluorescence. BF: bright field. (**c–d**) CCF4 cleavage efficiencies for DCs cultured in the absence or presence of LPS or α-tocopherol (α-Toc) (**c**) and for NOX2^KD^ DCs (**d**). NT: non-targeting siRNA. (**e–f**) Cell viability by the MTT assay of cells cultured with exogenous cytochrome C (Cyt C) and in presence or absence of α-tocopherol (α-Toc) (**e**) as well as for NOX2^KD^ DCs (**f**). Each donor is plotted in a different color. (**g**) Confocal micrographs of an unstimulated (no LPS) and stimulated (+LPS) DC expressing galectin-3-mAzami (green), incubated with OVA conjugated to Alexa fluor 647 (OVA-AF647; red). (**h**) Fluorescence intensities of galectin-3-mAzami thresholded on OVA-AF647, relative to cytosolic fluorescence intensity, in DCs in the presence or absence of LPS and α-Toc. Results show individual results for at least 3 donors. Scale bars, 10 μm.

We confirmed that lipid peroxidation could cause leakage of artificial membranes by measuring ROS-induced leakage of the 14 kDa model protein lysozyme from 400 nm-sized liposomes composed of (poly)unsaturated phospholipids. These liposomes were exposed to a combination of H_2_O_2_ and iron, which causes formation of hydroxyl radicals, lipid peroxidation and membrane leakage^18^. Leakage of lysozyme from the liposomes makes it accessible for trypsin digestion and the fraction of digested lysozyme hence correlates with liposome leakage. In agreement, the combination of H_2_O_2_ and iron caused leakage of lysozyme (~60%) as compared to the negative control in absence of ROS and the positive control with complete rupture of the liposomes by the detergent Triton X-100 (Fig. 2a). These results confirm the well-established notion that ROS-induced lipid peroxidation can trigger membrane leakage^20^,^21^,^22^. We then addressed whether NOX2-induced lipid peroxidation would also cause endosomal leakage in DCs.

Leakage of proteins from endosomes into the cytosol was measured by three assays. The first assay is based on the FRET sensor coumarin-cephalosporin-fluorescein (4)-acetoxymethyl (CCF4-AM)^4^,^8^. This probe accumulates in the cell and can be cleaved by the enzyme β-lactamase, which results in a loss of FRET between its coumarin (donor) and fluorescein (acceptor) fluorophores. We observed both CCF4 signal uniformly distributed in the cells and in punctate structures, indicating that CCF4 (and its cleavage products) localized partly in the cytosol and partly in intracellular compartments. Mammalian cells do not produce β-lactamase, but exogenous β-lactamase can be taken up by DCs and cleave CCF4 after β-lactamase escapes from endosomes into the cytosol or, conversely, after CCF4 influx into endosomes (Fig. 2b). CCF4 cleavage was promoted by activating the DCs with LPS, but could be blocked by the presence of α-tocopherol during β-lactamase uptake (Fig. 2c), demonstrating a role for lipid peroxidation in β-lactamase release from endosomes. α-Tocopherol protects cellular membranes from ROS-induced damage, not only by scavenging radicals, but also by promoting a negative curvature^34^. CCF4 cleavage was significantly reduced in human NOX2^KD^ DCs compared to controls (Fig. 2d). We could not perform endosomal leakage experiments with PAO, as this compound blocks endocytosis (Suppl. Fig. 2). Uptake was neither affected by α-tocopherol nor by knockdown of NOX2.

The second antigen leakage assay is based on the induction of apoptosis by exogenous cytochrome C. The presence of cytochrome C in the cytosol initiates apoptotic protease-activating factor 1 (Apaf-1) dependent apoptosis^4^,^8^,^35^. Leakage of exogenous cytochrome C from endosomes results in reduced cell viability, which can be measured by the MTT assay. α-Tocopherol was able to block cytochrome C-induced cell death, in accordance with our results with the CCF4 assay, supporting a role for NOX2-induced lipid peroxidation in cytochrome C escape from endosomes (Fig. 2e). RNAi knock-down of NOX2 even increased viability (compared to control cells without cytochrome C), which we attribute to reduced cell-damaging effects of ROS (Fig. 2f).

The third antigen leakage assay is based on the recruitment of galectin-3 to β-galactoside sugars, which are present on glycosylated proteins on the luminal, but not the cytosolic, side of endosomal membranes. In cells with intact endosomes, heterologously expressed galectin-3 tagged with the fluorescent protein mAzami is uniformly distributed in the cytosol of cells, but it clusters when it gains access to glycosylated moieties upon endosomal disruption^36^,^37^,^38^. As expected, LPS stimulation of DCs transfected with galectin-3-mAzami promoted the recruitment of galectin-3 to OVA-positive endosomes (Fig. 2g) and this could be blocked by inhibiting lipid peroxidation with α-tocopherol (Fig. 2h). Based on these data, we conclude that NOX2-induced lipid peroxidation can cause escape of antigen from endosomes into the cytosol.

### Lipid peroxidation promotes cross-presentation

**Figure 3:**
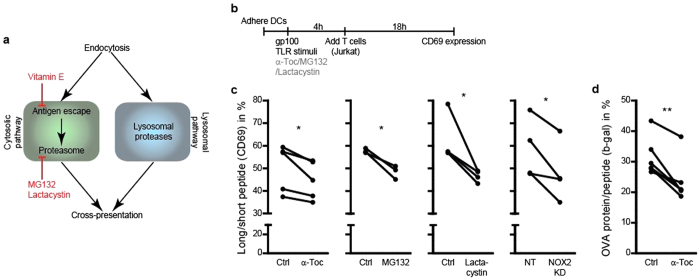
Lipid peroxidation promotes cross-presentation. (**a**) Scheme of the cytosolic and lysosomal pathways leading to antigen cross-presentation. (**b**) Time line of the Jurkat T cell activation assay with DCs presenting gp100. (**c**) Jurkat T cell activation assay with DCs incubated with α-tocopherol (α-Toc), MG132 or lactacystin and with NOX2^KD^ DCs after incubation with long (residues 272–300) or short peptide (residues 280–288). On the y-axes the percentages of CD69-positive T cells for long (residues 272–300) over short peptide are depicted. (**d**) B3Z T cell activation assay with BMDCs incubated with α-tocopherol (α-Toc) after incubation with complete OVA protein or OVA peptide (SIINFEKL). On the *y*-axis the percentages of β-galactosidase producing T cells for OVA protein over OVA peptide. Results show individual results for at least 3 donors. Raw FACS data, and full short and long peptide T cell activation controls are in Suppl. Figs 3 and 4.

Next, we addressed whether endosomal antigen escape by lipid peroxidation promotes antigen cross-presentation with use of two assays. We first used a well-established assay to measure cross-presentation based on the loading of DCs with part of the tumor antigen gp100 (residues 272–300; long peptide)^13^,^39^,^40^. A smaller fragment of this long peptide (residues 280–288; short peptide) can be derived by lysosomal proteases (lysosomal pathway) or by the proteasome (cytosolic pathway) and cross-presented to a Jurkat T cell line heterologously expressing a receptor specific for MHC class I (HLA-A2) carrying gp100 short peptide (Fig. 3a). Upon activation of this receptor, these Jurkat T cells increase expression of the activation marker CD69 on their cell surface, which can be measured by flow cytometry (Fig. 3b and Suppl. Fig. 3a). Under our experimental conditions, the maximum percentage of activated Jurkat T cells was ~55%, as determined by incubating DCs with an excess of short peptide that can exchange with endogenous peptide in MHC class I (Suppl. Fig. 3b–d). Importantly, the Jurkat T cell assay provides a direct measure for cross-presentation as Jurkat activation is independent of the co-stimulatory factors that are required for activation of naive T cells (e.g. CD80, CD86, IL-12, IFNγ).

We started by confirming the known role of NOX2 in promoting cross-presentation^10^,^11^,^13^,^14^,^41^. The activation of Jurkat T-cells by NOX2^KD^ DCs was significantly reduced by ~20% compared to control, similar to the inhibitions that we observed by treatment with the proteasome inhibitors MG132 and lactacystin (Fig. 3c). As antigens such as gp100 can be cross-presented via both proteasome-dependent (i.e. cytosolic) and -independent (i.e. lysosomal) pathways^42^, our results indicate that, under our experimental conditions, about 20% of gp100 is cross-presented via the cytosolic pathway and that this pathway depends on NOX2. Importantly, selectively inhibiting lipid peroxidation with α-tocopherol during antigen uptake by DCs also significantly reduced Jurkat T cell activation (Fig. 3c).

As a second assay for cross-presentation, we performed a T cell activation assay with mouse BMDCs loaded with the model antigen OVA. OVA can also be partly processed in the cytosol by the proteasome and cross-presented in MHC class I to B3Z cells expressing a specific T cell receptor^4^,^10^,^11^,^41^. Engagement of this receptor results in the production of β-galactosidase by these B3Z cells, which can be measured by colorimetric assays (Suppl. Fig. 4a). Similar to the Jurkat T cell assay, B3Z cells allow to directly measure cross-presentation as B3Z activation is independent of the co-stimulatory factors that are required for activation of naive T cells. In accordance with our results from the gp100 assay with human DCs, α-tocopherol treatment decreased activation of B3Z cells by ∼25% using OVA (Fig. 3d) and this was not changed using the specific OVA antigenic peptide (Suppl. Fig. 4b). We were not able to perform the B3Z assay in combination with MG132 and lactacystein due to high toxicity of these proteasome inhibitors (Suppl. Fig. 4c). These data are in accordance with our hypothesis that lipid peroxidation triggers endosomal antigen release and this promotes cross-presentation. We then performed optogenetic experiments to more directly confirm this causal relationship.

### Light-triggering lipid peroxidation causes antigen release

**Figure 4:**
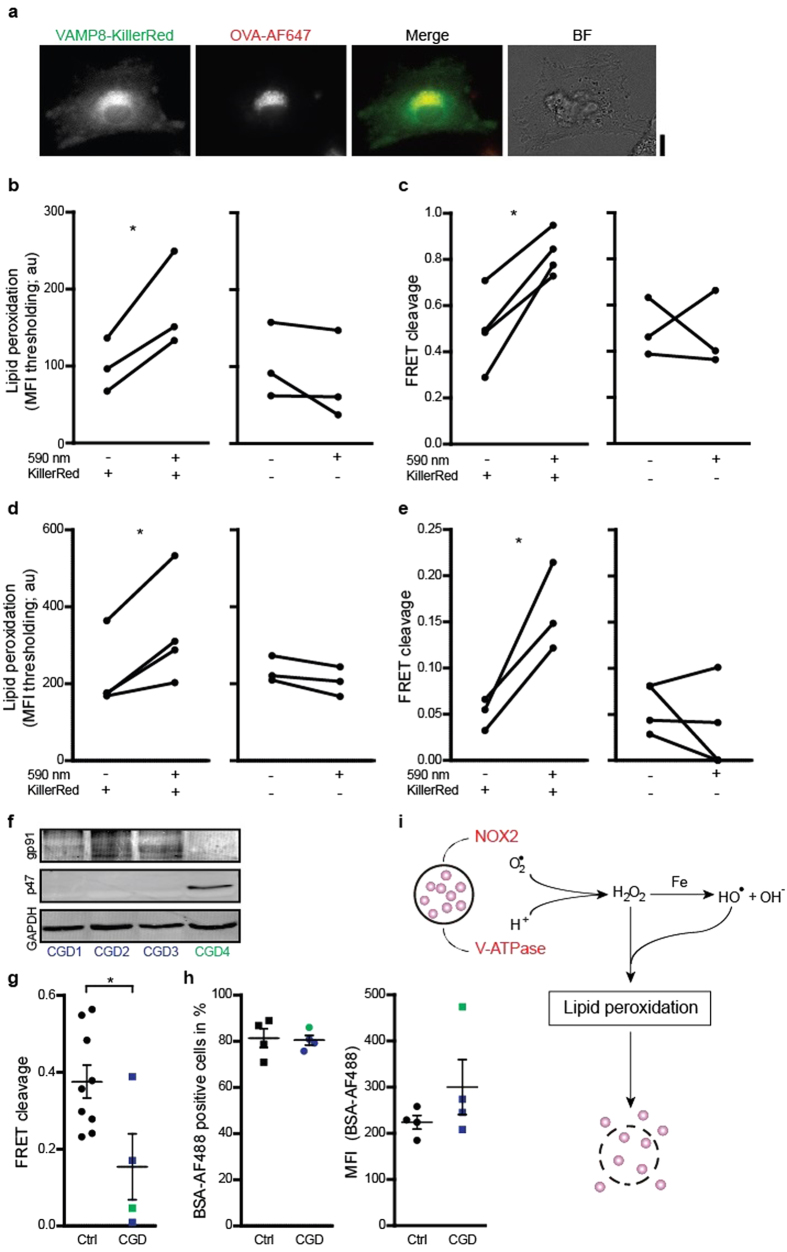
Direct induction of endosomal antigen release and impaired release in CGD. (**a**) Confocal micrographs of a DC expressing VAMP8-KillerRed (green) and incubated with OVA conjugated to Alexa fluor 647 (OVA-AF647; red). BF: bright field. Scale bar, 10 μm. (**b**) Linoleamide alkyne lipid peroxidation assay as in fig. 1i, but now for exposed or non-exposed DCs with or without expression of KillerRed. (**c**) CCF4 endosomal antigen leakage assay as in fig. 2b, but now for exposed or non-exposed DCs with or without expression of KillerRed. (**d–e**) Similar as panels (**b,c**) but now for HEK293T cells. (**f**) p47phox and gp91phox expression in lymphocytes from three p47phox-/- (CGD1-3; blue) and one gp91phox-/- (CGD4; green) CGD patients by Western blot (GAPDH: loading control). (**g**) CCF4 endosomal antigen release assay as in fig. 2b, but now with DCs derived from monocytes from the CGD patients from panel (**d**) (Ctrl: DCs from healthy donors). (**h**) Uptake of BSA conjugated to Alexa fluor 488 (BSA-AF488) by DCs of CGD patients, presented as percentage BSA-AF488-positive cells and MFI. Results show individual results of at least 3 donors/experiments. (**i**) Model scheme of NOX2-induced antigen leakage from endosomes. Superoxide anions produced by NOX2 and protons provided by the V-ATPase form H_2_O_2_ and hydroxyl radicals (Fenton reaction). These ROS induce lipid peroxidation, which disrupts endosomal membranes and causes the release of antigen (purple spheres) into the cytosol.

We developed an optogenetic approach to light-trigger lipid peroxidation specifically in endosomes of DCs. This system is based on the fluorescent protein KillerRed, which generates singlet oxygen and superoxide anion radicals upon exposure to orange light (at 590 nm)^43^. KillerRed was targeted to the antigen processing compartment by genetic fusion to the endosomal SNARE protein VAMP8 (Fig. 4a), which traffics gp91phox to phagosomes^41^. DCs expressing VAMP8-KillerRed were exposed to light by culturing directly under an orange LED array. These exposure conditions did not result in decreased DC viability, but did result in an almost 2-fold increased lipid peroxidation in endosomes compared to unexposed cells and to cells without expression of KillerRed as determined by the LAA assay (Fig. 4b). We could not use the Bodipy581/591-C11 assay in these experiments, because of spectral overlap of this probe with KillerRed. As expected, KillerRed activation also resulted in more antigen leakage from endosomes, as we observed an almost 2-fold increase of CCF4 cleavage efficiency compared to unexposed cells and to cells without expression of KillerRed (Fig. 4c). In these LAA and CCF4 experiments, we identified KillerRed-positive cells by residual (i.e. unbleached) fluorescence. Lipid peroxidation and the release of antigen upon VAMP8-KillerRed exposure was not specific for immune cells, as we observed similar results with the embryonic kidney cell line HEK293T (Fig. 4d–e) and with the human melanoma cell line MEL624. Although HEK293T cells are HLA-A2 positive^44^, we did not observe cross-presentation by these cells with the Jurkat T cell assay, perhaps because of a lack of components required for subsequent antigen processing, such as the immunoproteasome^45^,^46^. Together, these data prove that lipid peroxidation causes antigen leakage from endosomes.

### Endosomal antigen leakage is reduced in CGD patients

In the final set of experiments, we measured endosomal leakage in DCs isolated from blood of patients suffering from chronic granulomatous disease (CGD). CGD patients have a genetic mutation resulting in dysfunctional NOX2 and increased susceptibility to infectious diseases^15^. Cross-presentation is also impaired in DCs of CGD patients^13^,^47^ and this has been linked to the higher risk of developing autoimmune diseases^48^ and impaired antifungal protection^47^. We performed the CCF4 assay on DCs from three CGD patients with mutated p47phox (a cytosolic component of NOX2) and one patient with mutated membrane-bound gp91phox (Fig. 4f). In agreement with our experiments with NOX2^KD^, CCF4 cleavage was substantially reduced in DCs from CGD patients compared to healthy individuals (Fig. 4g), whereas control experiments with BSA conjugated to Alexa fluor 488 showed that antigen uptake was not changed significantly (Fig. 4h). This result demonstrates that release of antigen from endosomes is impaired in CGD patients, further supporting a role of NOX2 in this process.

## Discussion

The pivotal role of NOX2 in facilitating cross-presentation is well-established^10^,^11^,^12^,^13^,^14^,^41^. Although it is clear that NOX2 preserves antigen by inhibiting endo/lysosomal proteases via oxidation^14^,^16^,^17^ and delayed acidification^10^,^11^,^12^, the direct mechanistic link between NOX2-produced ROS and cross-presentation was unclear^13^. We now show that NOX2-produced ROS induce lipid peroxidation in endosomal membranes and thereby can cause antigen leakage from the endosomal lumen into the cytosol (Fig. 4i). These findings are in line with the reduced lipid peroxidation observed upon inhibition of NOX2 in murine neurons and microglia^49^ and with the role of ROS in cathepsin release from lysosomes^50^,^51^. Our findings explain how neutrophils, which have very high NOX2 activity during the oxidative burst, can be efficient antigen cross-presenting cells^52^. Our findings also corroborate the suggestion that cross-presentation is facilitated by pathogens that actively disrupt the phagosomes of their host-cells^53^. This disruption allows the microbes access to nutrients from the cytosol of the host cell and this is required for their replication, but could also enable their detection by the immune system.

Our study also provides a new mechanistic link of the well-established positive correlation between cross-presentation and autophagy^54^. NOX2-generated ROS are necessary for the induction of autophagy by phagosomal recruitment of the autophagy protein LC3^55^,^56^. This LC3 recruitment is compromised in CGD patients resulting in autophagic defects, which underlie the pathogenesis of granulomatous colitis^56^. The recruitment of LC3 to phagosomes can be blocked with α-tocopherol^55^, suggesting the involvement of intracellular scavenger receptors that recognize endosomal products of lipid peroxidation. Our results now show that at this point the integrity of the phagosomal membrane can be compromised, resulting in the leakage of antigen into the cytosolic MHC class I pathway and hence promoting cross-presentation.

Another topic of intense study is the role of ROS in cancer and inflammation^57^. ROS are well-known to act as secondary messengers for activation of immune cells^58^ and regulate for instance T cell activation by DCs^59^. In this study, we have identified an additional mechanism of regulation of the immune function by ROS. Our results show that NOX2-induced ROS can cause lipid peroxidation, which in turn can result in release of (tumor) antigen into the cytosol, where it becomes accessible to the proteasome. Cross-presentation is hence facilitated by oxidation of endosomal lipids and this is a new cellular role for ROS. As already suggested previously^3^,^9^,^27^, the aspecific leakage of endosomal compartments explains how large, folded and/or posttranslationally modified proteins as well as nucleic acids and dextrans can reach the cytosol. Thus, although our results do not exclude roles for specific protein channels such as Sec61^5^,^6^,^7^,^8^, lipid peroxidation provides an, at least complementary, mechanism by which antigens escape from endosomes for cross-presentation by DCs.

The release of antigen from endosomes by lipid peroxidation may be used as a novel strategy to combat disease. Augmenting ROS specifically in endosomes or phagosomes of DCs by means of radical inducing molecules may help to evoke a CD8 + T cell response against cancer and infectious disease. Such an approach might well be feasible, given that the generation of ROS in endosomes of target cells is already exploited for delivery of siRNA in the gene therapy field^23^,^24^,^25^,^26^. Thus, our results underscore that approaches to target ROS in endosomes of antigen presenting cells might be a promising therapeutic strategy for cancer and (auto)immune diseases.

## Methods

### Cells and transfection

Human DCs were derived from peripheral blood monocytes (PBMCs) obtained from buffy coats of healthy individuals (informed consent obtained from all individuals) according to institutional guidelines and as described previously^60^. Briefly, after purification on a Ficoll-gradient, monocytes were differentiated into immature DCs by culturing 6 days at 37 °C (5% CO_2_) in the presence of 300 U/ml IL-4 and 450 U/ml granulocyte macrophage colony-stimulating factor (GM-CSF) in complete RPMI-1640 medium (Gibco by Thermo Fisher) containing 10% fetal bovine serum (FBS; Greiner Bio-one), 2 mM UltraGlutamine (Lonza) and 1% Antibiotic-Antimytotic (AA; Gibco by Thermo Fisher). CGD DCs were derived from PBMCs obtained from blood of three *p47phox-/-* and one *gp91phox-/-* patients^61^ using the same protocol. All CGD patients were between the age of 5 to 13 years and free from any infectious or inflammatory diseases. HEK293T cells were cultured in DMEM (Gibco by Thermo Fisher) containing 10% FBS, 1% non-essential amino acids and 0.5% AA. Jurkat E6.1 cells expressing CD8 + T cell receptors that recognize gp100-peptide(280–288) in the context of HLA-A2 were used for the T cell activation assay with human DCs. They were cultured in RPMI-1640 containing 10% FBS, 2 mM UltraGlutamine and 0.5% AA.

Mouse bone marrow derived dendritic cells (BMDCs) were obtained from healthy C57BL/6 mice. BMDCs were differentiated by culturing 7 days in the presence of 20 ng/ml mouse GM-CSF in RPMI-1640 containing 10% FBS, 2 mM UltraGlutamine, 1% AA and 28 μM β-mercaptoethanol. B3Z T cell hybridoma cells containing a lacZ construct inducible by activation of its T cell receptor specific for OVA peptide in the context of H-2 Kb^62^ were used for the T cell activation assay with BMDCs. B3Z cells were cultured in the presence of 1 mM hygromycin B in IMDM containing 5% FBS, 2 mM UltraGlutamine, 1% AA and 28 μM β-mercaptoethanol. All experiments with human samples were conducted in accordance with the relevant guidelines. Patient tissue and samples were obtained in accordance with the Declaration of Helsinki and approved consent was obtained from all patients or their legal representatives. All experiments were approved by the ethics committee from Radboud University Medical Center.

For NOX2^KD^, DCs were electroporated with siRNA against NOX2 subunit *gp91phox* (CCGAGUCAAUAAUUCUGAUCCUUAU; Thermo Fisher) or with irrelevant ON-TARGET plus Non-Targeting (NT) siRNA#1 (Dharmacon) using a Neon Transfection System (Thermo Fisher; 1,000 V, 40 ms, 2 pulses). After electroporation, cells were transferred to serum-free RPMI medium without AA and phenol red. After 3 hours, this medium was replaced by complete RPMI medium and cells were cultured for 48 hours to achieve maximal knock-down, as judged by Western analysis. Proteins were detected with mouse monoclonal antibody anti-NOX2/gp91phox (ab80897, Abcam) and rabbit polyclonal antibody anti-p47phox (sc-14015, Santa Cruz) (all at 1:500 dilution (v/v)) in combination with goat anti-rabbit or goat anti-mouse conjugated with IRDye 680 or 800 (all LI-COR) as secondary antibodies (1:5,000 dilution (v/v)).

The construct coding for VAMP8-pKillerRed was made by standard cloning procedures. First mouse VAMP8 with restriction sites XhoI and BamHI was generated by PCR, cut and ligated into pmCherry-N1. Next, mCherry was replaced with KillerRed^43^ via BamHI/NotI restriction sites. KillerRed-N1 was a gift from Michael Davidson (Addgene plasmid # 54636). DCs were electroporated with the VAMP8-KillerRed construct as described^63^. HEK293T cells were transfected using lipofectamine (Thermo Fisher), following the procedures of the manufacturer. DCs and HEK293T cells were cultured for 16 hours to achieve highest expression levels of VAMP8-KillerRed. For exposure, DCs and HEK293T cells expressing VAMP8-KillerRed were cultured for 0.5 – 4 hr directly under a 109 mW LED array at 590 nm (Thorlabs).

### Lysozyme leakage assay

A mixture composed of 25 mg 1,2-dioleoyl-sn-glycero-3-phosphocholine, 25 mg 1-stearoyl-2-arachidonoyl-sn-glycero-3-phosphocholine, 5 mg DOPS 1,2-dioleoyl-sn-glycero-3-phospho-L-serine and 5 mg cholesterol (all Avanti) in chloroform was dried in a stream of N_2_ followed by vacuum. After drying, lipids were resuspended in 1 ml PBS containing 5% (w/v) sodium cholate and 7 mg/ml lysozyme (from chicken egg white; Sigma). The emulsion was dialyzed (7 kDa MWCO) and extruded through a polycarbonate filter with 400 nm pore size. Non-encapsulated lysozyme was removed by gel filtration (Sephadex-G150). The liposomes were incubated with 200 mM H_2_O_2_ and 100 μM freshly prepared Fe(II)SO_4_ (Sigma) for 3 hours at room temperature (RT) followed by incubation with 50 mg/ml trypsin for 4 hours at 37 °C. Positive control experiments were performed with 0.2% Triton X-100. Samples were analyzed by 15% SDS-PAGE and stained with Coomassie Brilliant Blue R250 (CBB).

### ROS and lipid peroxidation assays

For the Amplex Red assay, cells were incubated for 2–4 hours in a 96 well plate (100,000 cells/well) in serum-free RPMI with 2.2 mM OVA (Sigma) and with or without 1 μg/ml LPS (Sigma). After incubation, the medium was changed for RPMI without phenol red containing 50 μM Amplex UltraRed reagent (Thermo Fisher) and 3 U/ml horseradish peroxidase (Sigma) and the fluorescence of resofurin was measured every 15 minutes for 2 hours of subsequent incubation.

For the Bodipy581/591-C11 assay, DCs were loaded with 2.2 mM OVA and cultured in the presence of 1 μM Bodipy581/591-C11 (4,4-difluoro-5-(4-phenyl-1,3-butadienyl)-4-bora-3a,4a-diaza-s-indacene-3-undecanoic acid; Thermo Fisher) in serum-free RPMI for 60 minutes at RT. 1 μg/ml LPS, 100 μM α-tocopherol (Sigma) and/or 1 μM phenylarsine oxide (PAO, Sigma) were added together with Bodipy581/591-C11. After incubation, the Bodipy581/591-C11 fluorescence was measured by flow cytometry (excitation: 488 nm; emission: 530/30 nm; FACSCaliber, BD biosciences). The signal before incubation was subtracted for background correction.

The LAA Click chemistry experiments were performed following the manufactures protocol (C10445, Thermo Fisher) with some modifications. DCs were loaded with 55.5 μM OVA-Alexa fluor 647 and 1 μg/ml LPS as adjuvant and cultured in the presence of 50 μM Click-iT LAA solution and 100 μM α-tocopherol in serum-free RPMI for 1 hour at 37 °C on 12 mm coverslips in 24 wells plates. KillerRed-transfected DCs were exposed during incubation as described above. After culturing, the cells were washed, fixed with 4% PFA, permeabilized with 0.5% TX100 and blocked with 1% BSA (all diluted in PBS and incubated at RT). Coverslips were incubated for 30 minutes at RT protected from light with Click-iT reaction cocktail containing Cu(II)SO_4_ and Alexa fluor 488 azide. Finally, the cells were washed and embedded in mounting medium containing 0.01% (v/v) Trolox and 68% (v/v) glycerol. Imaging was done with a Leica SP8 confocal microscope with a 63 × 1.20 NA water immersion objective. For analysis of the green fluorescent signal, the endosomes were identified by setting a threshold on the signal of the OVA-Alexa fluor 647 channel.

### Antigen uptake and leakage assays

For the antigen uptake experiments, DCs were incubated with 100 μg/ml BSA-Alexa fluor 488 (Thermo Fisher) and 1 μg/ml LPS for 60 minutes in the presence or absence of 500 μM α-tocopherol or 1 μM PAO. After washing the cells, the percentage of BSA-AF488 positive cells was measured by flow cytometry.

For the cytochrome C experiments^35^, DCs were incubated with 10 mg/ml cytochrome C (from equine heart; Sigma), 1 μg/ml LPS and/or 500 μM α-tocopherol in complete RPMI for 20 hours at 37 °C. After incubation, the medium was removed and 0.5 mg/ml MTT (3-(4,5-dimethylthiazol-2-yl)-2,5-diphenyltetrazolium bromide) was added to each sample. After 1–4 hours incubation, lysis buffer containing 90% iso-propanol, 0.0125% SDS and 4% 1 M HCl was added and absorbance was measured at 595 nm to determine cell death.

The CCF4/β-lactamase assay^36^ was performed in Willco petri dishes according to the protocol of the manufacturer (K1095, Thermo Fisher). Briefly, DCs were incubated with 1 μM Substrate Loading Solution, 1 μg/ml LPS as adjuvant and if applicable 500 μM α-tocopherol in Live Cell Imaging Medium (Thermo Fisher) for 90 minutes at 37 °C. After 90 minutes incubation time, DCs were washed and incubated with 1 mg/ml β-lactamase (Sigma). In addition, 1 μg/ml LPS and/or 500 μM α-tocopherol were included together with the β-lactamase if these were added during the first incubation step. The second incubation step was done in Live Cell Imaging Medium for 3 hours at 37 °C. The KillerRed-transfected DCs were exposed or shielded from light during the last 30 minutes of incubation. Cells were washed again and imaged with a Leica SP8 confocal microscope and a 63 × 1.20 NA water immersion objective (405 nm excitation; 419–458 nm blue and 504–600 nm green emission of the coumarin donor and fluorescein acceptor fluorophores, respectively). The FRET efficiencies *F* were calculated as the ratio of the green over the blue fluorescence intensities. The FRET efficiency of control cells in absence of β-lactamase *F*_neg_ was used as a negative control. The FRET cleavage *C* was then calculated as (Eqn 1):


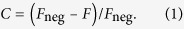


For the galectin-3 experiments, galectin-3-mAzami was cloned from mAG-GAL3^38^ into the NotI and PspOMI sites of pCDNA6-myc His B (Thermo Fisher). mAG-GAL3 was a gift from Niels Geijsen (Addgene plasmid # 62734). DCs were transfected with galectin-3-mAzami, following the same procedure as for Vamp8-KillerRed. After electroporation, the DCs were cultured for 16 h on coverslips and subsequently incubated with OVA conjugated to Alexa fluor 647 for 1 h in the presence or absence of 1 μg/ml LPS and/or 500 μM α-tocopherol. After incubation, cells were fixed with 4% PFA and embedded in mounting medium containing 0.01% (v/v) Trolox and 68% (v/v) glycerol. Imaging was done with a Leica DM6000 epi-fluorescence microscope fitted with a 63 × 1.40 NA oil immersion objective and appropriate filters for mAzami (excitation: BP470/40; dichroic: LP495; emission: BP524/50) and Alexa fluor 647 (excitation: BP620/60; dichroic: LP660; emission: BP700/75). For analysis, the fluorescence intensities of galectin-3-mAzami from individual releasing endosomes, identified by the OVA-Alexa fluor 647 channel, were corrected for the cytosolic fluorescence intensities similar as described^64^.

### pH measurements

For the labeling of OVA with pHrodo, a stock solution of 9 mM pHrodo Green STP ester (Thermo Fisher) in DMSO was mixed with 10 volumes of 10 mg/ml OVA and incubated for 2 h at RT. DCs were pulsed with 1 mM pHrodo-conjugated OVA and 1 μg/ml LPS in the presence or absence of 500 μM α-tocopherol for 15 minutes, followed by a chase of 150 minutes. The fluorescence intensities of pHrodo was measured by flow cytometry at 20, 40, 60, 90, 120 and 150 minutes after starting with the chase (excitation: 488 nm; emission: 530/30 nm). pH was calibrated by measuring the fluorescence of pHrodo in a standard solution series of known pH.

For the SNARF-1 experiments, DCs adhered to glass bottom microdishes were washed and incubated for 30 minutes with 1 mg/ml SNARF-1 dextran 10,000 MW (D3303, Thermo Fisher) with or without 500 μM α-tocopherol or 1 μM PAO. DCs were imaged with a Leica SP8 confocal microscope and a 63 × 1.20 NA water immersion objective (540 nm excitation; 550–600 nm green and 620–700 nm red emission peaks of SNARF-1). The pH calibration curve was created by imaging SNARF-1 dextran-containing DCs that were incubated with buffers of different pH (10 mM phosphate buffer, 150 mM NaCl) and 0.003% Triton-X100.

### Cross-presentation assays

For the human T cell activation assay, HLA-A2 positive DCs (70,000 cells/well) were incubated for 1 hour at 37 °C in X-vivo medium (Lonza) containing 2% human serum in 96 wells plates. Cells were then washed and incubated with gp100 short peptide (10 μM gp100(280–288); GenScript) or long peptide (100 μM gp100(272–300); JPT) in combination with T cell receptor stimulation mix (4 μg/ml R-848 and poly(I:C); Enzo Life Sciences) for 4 hours in X-vivo medium at 37 °C. If applicable 500 μM α-tocopherol, 10 μM MG132 (Sigma) or 10 μM lactacystin (Sigma) were also added during this time step. The KillerRed-transfected DCs were exposed or shielded from light during antigen uptake. After incubation, cells were washed and resuspended in X-vivo medium containing Jurkat E6.1 cells (100,000 cells/well) carrying a specific T cell receptor recognizing HLA-A2 with gp100(280–288)^65^ and co-cultured for 18 hours at 37 °C. T cells were stained with αCD69-FITC (1:10, BD biosciences), αCD3-APC (1:10, eBiosciences) and 7AAD viability staining (1:14, Biolegend) or Propidium Iodide (PI; 0.5 μg/ml) and analyzed with a FACSCalibur (BD biosciences). CD69 expression to measure T cell activation was analyzed using FlowJo software and Prism 5. Briefly, cells were gated on viability (7AAD or PI), followed by gating for CD3 staining, and CD69 expression was measured. T cell activation was calculated as the ratio of CD69 positive cells stimulated with long over stimulation with short peptide.

For the T cells activation assay with mouse cells, BMDCs (from H-2 Kb positive C57BL/6 mice; 80,000 cells/well) were incubated with 80 μg/ml OVA protein, 5 ng/ml OVA peptide (residues 257–264; SIINFEKL), 1 μg/ml LPS and if applicable 500 μM α-tocopherol in complete RPMI-1640 for 30 minutes (OVA peptide) or 5 hours (OVA protein) at 37 °C. After incubation, cells were washed and incubated in complete RPMI-1640 containing B3Z cells (80,000 cells/well) for 18 hours. Supernatant was removed and cells were incubated with Z-buffer containing 10 mM DTT (MP Biomedicals), 9 mM MgCl_2_, 0.15 mM CPRG (Calbiochem) and 0.125% NP40 (Sigma) for 1–5 hours at 37 °C. Absorbance was measured at 595 nm. Total T cell activation was calculated as the ratio of the absorbances of cells stimulated with the full-length OVA protein over stimulation with the OVA peptide.

### Statistical analysis

All experiments were assessed using paired Student t tests, except for the data with CGD patient material where an unpaired Student t test was used. Two-sided *P* values <0.05 were considered to be statistically significant (*p < 0.05, **p < 0.01, ***p < 0.001).

## Additional Information

**How to cite this article**: Dingjan, I. *et al.* Lipid peroxidation causes endosomal antigen release for cross-presentation. *Sci. Rep.* **6**, 22064; doi: 10.1038/srep22064 (2016).

## Supplementary Material

Supplementary Information
