# Supplementary material for: Lipid peroxidation causes endosomal antigen release for cross-presentation
Source: Sci Rep. 2016 Feb 24;6:22064. doi: 10.1038/srep22064 (PMC4764948; doi:10.1038/srep22064)
Supplement: Supplementary Information [file srep22064-s1.pdf]

Supplementary information

Lipid peroxidation causes endosomal antigen release for cross-presentation

Ilse Dingjan[1], Daniëlle RJ Verboogen[1], Laurent M Paardekooper[1], Natalia H Revelo[1], Simone P Sittig[1], Linda J Visser[1], Gabriele Fischer von Mollard[2], Stefanie SV Henriet[3], Carl G Figdor[1], Martin ter Beest[1], Geert van den Bogaart[1,4]

Affiliations

[1]Department of Tumor Immunology, Radboud Institute for Molecular Life Sciences, Radboud University Medical Center, Nijmegen, 6525 GA, the Netherlands.

[2]Department of Chemistry, Bielefeld University, Bielefeld, 33501, Germany.

[3]Department of Pediatrics, Radboud University Medical Center, Nijmegen, 6525 GA, the Netherlands.

[4]**Corresponding author:** Geert van den Bogaart; Radboud University, Geert Grooteplein 26-28, 6525 GA Nijmegen; +31(0)243613662; Geert.vandenBogaart@RadboudUMC.nl

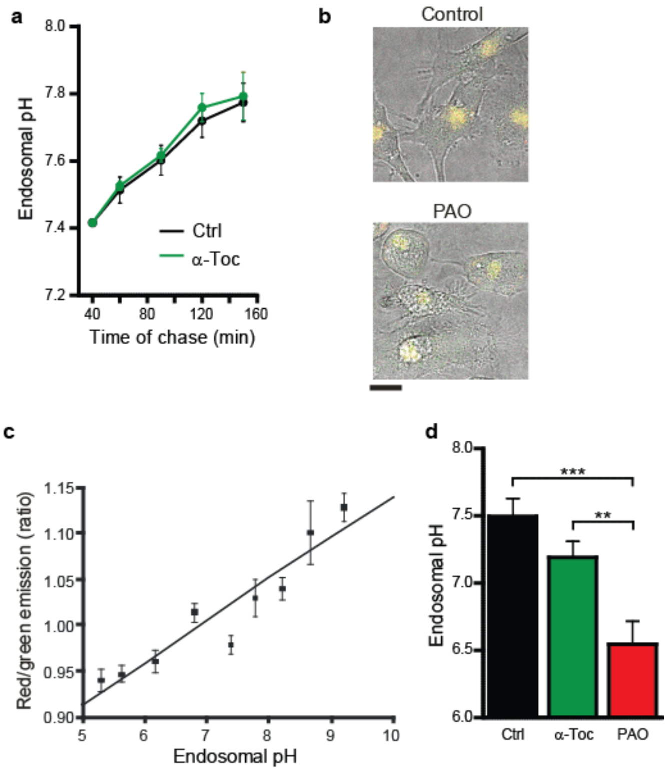

Supplementary Fig. 1: Endosomal pH is unaffected by α-tocopherol. (a) Endosomal pH determined with the intensiometric probe pHrodo Green linked to OVA for DCs incubated with or without α-tocopherol. The pH was calibrated by measuring the fluorescence of pHrodo Green in buffers of known pH. **(b−d)** Intensiometric probes are prone to artifacts caused by ROS-induced oxidation and we therefore confirmed the results from panel a with the ratiometric probe SNARF-1 conjugated to dextran. **(b)** Confocal microscope images of DCs with SNARF-1 with and without PAO. PAO results in endosomal acidification apparent from a green shift of the fluorescence of SNARF-1. Scale bar, 10 µm. **(c)** The pH calibration curve was created by imaging cells after uptake of SNARF-1-labeled dextran and incubation with buffers of different pH and 0.003% Triton-X100. The curve was fitted with a standard reaction model with pKa = 7.3 for SNARF-1 (at 37°C; Life Technologies; solid line). **(d)** Average of endosomal pH of > 100 cells (pooled from 3 independent experiments) within 30–60 min after SNARF-1 uptake. PAO resulted in acidification of endosomal compartments. Results show mean ± S.E.M of 3 donors.

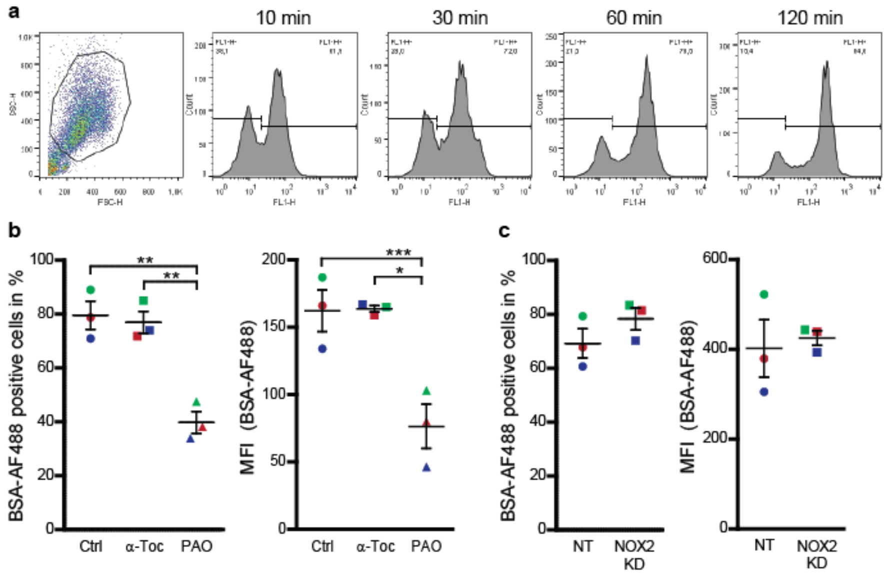

Supplementary Fig. 2: Controls for the endosomal leakage experiments. (a) Representative FACS data for uptake experiments of BSA conjugated to Alexa fluor 488 (BSA-AF488) by DCs. Left dot plot: side (SSC) and forward (FSC) scatter plot for gating of DCs. Right histograms: fluorescence distributions of BSA-AF488 signal at the indicated times after begin of uptake. **(b-c)** Uptake of BSA conjugated to Alexa fluor 488 (BSA-AF488) was measured after 60 min for DCs cultured in the absence (Ctrl) or presence of α-tocopherol (α-Toc) or PAO **(b)** as well as for NOX2^{KD} DCs **(c)**. NT: non-targeting siRNA control. PAO decreased uptake of BSA by ~50%. Results show results of 3 donors (indicated with different colors).

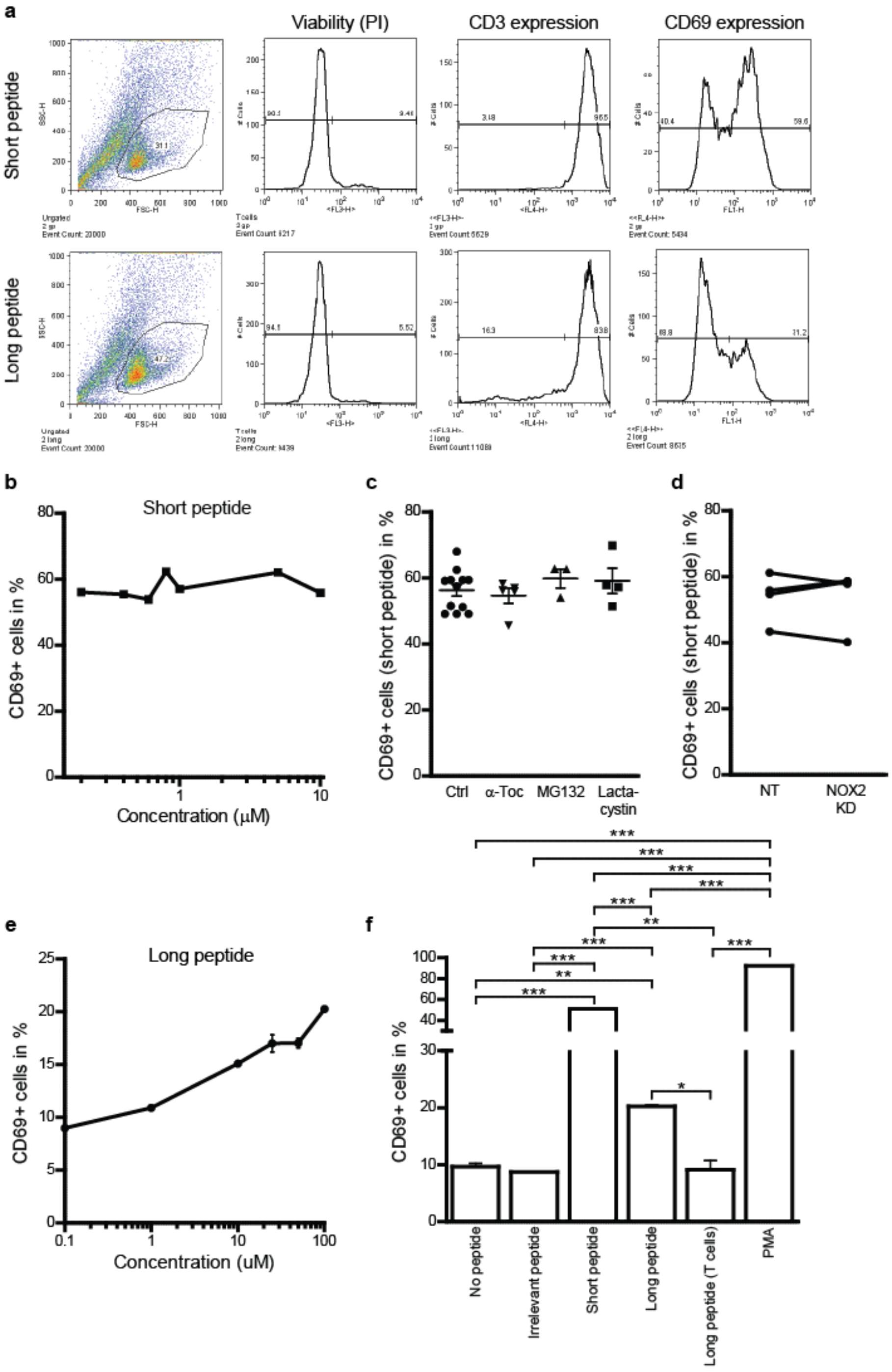

Supplementary Fig. 3: Short and long peptide controls for Jurkat T cell activation. (a) Representative FACS data for analysis of CD69-positive T cells. Jurkat T cells were selected based on scatter (left dot plot) and gating on PI-negative, CD3-positive cells (middle histograms). Right histogram: CD69 intensity distribution for Jurkat T cells.

(b) Activation of Jurkat T cells by DCs incubated with a concentration range of short peptide (gp100 residues 280–288). For all concentrations, we observed a maximum activation of ~55% CD69-positive T cells with short peptide. **(c-d)** Jurkat T cells activation assay with DCs incubated with α-tocopherol (α-Toc), MG132 or lactacystein **(c)** and with NOX2^{KD} DCs **(d)** after incubation with short peptide. **(e)** Similar to panel b, but now for a concentration range of long peptide (residues 272-300). **(f)** CD69 expression on T cells after incubation with DCs treated with irrelevant, short or long peptide. As a negative control, T cells were incubated with long peptide in absence of DCs. As a positive control, T cells were activated by PMA. Results show mean ± spread of 2 donors.

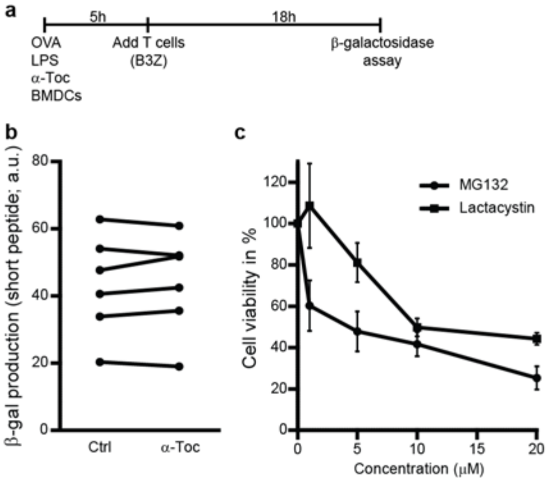

Supplementary Fig. 4: B3Z T cell activation assay with mouse BMDCs. (a) Time line of the B3Z T cell activation assay with BMDCs presenting OVA. **(b)** B3Z T cell activation assay with BMDCs incubated with α-tocopherol (α-Toc) after incubation with 5 ng/ml OVA peptide (SIINFEKL). **(c)** Toxicity of proteasome inhibitors MG132 and lactacystin. BMDCs were treated with different concentrations of MG132 and lactacystin for 4 h at 37°C. After incubation, the medium was removed and 0.5 mg/ml MTT was added to each sample. After 1-4 hours incubation, lysis buffer was added and absorbance was measured at 595 nm to determine cell viability. Results show average ± SEM of at least 3 experiments.
